# Real-Time Functional Magnetic Resonance Imaging Neurofeedback for the Relief of Distressing Auditory-Verbal Hallucinations: Methodological and Empirical Advances

**DOI:** 10.1093/schbul/sbaa103

**Published:** 2020-08-02

**Authors:** Clara Humpston, Jane Garrison, Natasza Orlov, André Aleman, Renaud Jardri, Charles Fernyhough, Paul Allen

**Affiliations:** 1 Institute for Mental Health, School of Psychology, University of Birmingham, Birmingham, UK; 2 Institute of Psychiatry, Psychology and Neuroscience, King’s College London, London, UK; 3 Department of Psychology, University of Cambridge, Cambridge, UK; 4 Athinoula A. Martinos Center for Biomedical Imaging, Massachusetts General Hospital, Boston, MA; 5 Department of Radiology, Xuanwu Hospital, Capital Medical University, Beijing, China; 6 Precision Brain Imaging Lab, Medical University of South Carolina, Charleston, SC; 7 Faculty of Medical Sciences, University of Groningen, AB Groningen, The Netherlands; 8 University of Lille, INSERM, CHU Lille, Lille Neuroscience and Cognition Centre (U-1172), Plasticity and Subjectivity (PSY) Team, CURE Platform, Lille, France; 9 Department of Psychology, Durham University, Durham, UK; 10 Department of Psychology, University of Roehampton, London, UK

**Keywords:** neurofeedback, hallucinations, schizophrenia, psychotic experiences, neuroimaging

## Abstract

Auditory-verbal hallucinations (AVH) are often associated with high levels of distress and disability in individuals with schizophrenia-spectrum disorders. In around 30% of individuals with distressing AVH and diagnosed with schizophrenia, traditional antipsychotic drugs have little or no effect. Thus, it is important to develop mechanistic models of AVH to inform new treatments. Recently a small number of studies have begun to explore the use of real-time functional magnetic resonance imaging neurofeedback (rtfMRI-NF) for the treatment of AVH in individuals with schizophrenia. rtfMRI-NF protocols have been developed to provide feedback about brain activation in real time to enable participants to progressively achieve voluntary control over their brain activity. We offer a conceptual review of the background and general features of neurofeedback procedures before summarizing and evaluating existing mechanistic models of AVH to identify feasible neural targets for the application of rtfMRI-NF as a potential treatment. We consider methodological issues, including the choice of localizers and practicalities in logistics when setting up neurofeedback procedures in a clinical setting. We discuss clinical considerations relating to the use of rtfMRI-NF for AVH in individuals distressed by their experiences and put forward a number of questions and recommendations about best practice. Lastly, we conclude by offering suggestions for new avenues for neurofeedback methodology and mechanistic targets in relation to the research and treatment of AVH.

## Introduction

Real-time functional magnetic resonance imaging neurofeedback (rtfMRI-NF) allows individuals to monitor and self-regulate their own brain activity by measuring this activity and feeding it back to the participant so that they can progressively subject it to voluntary control. Historically, the most commonly used neurofeedback methods employed electroencephalogram (EEG), with the focus shifting only relatively recently toward rtfMRI-NF. rtfMRI-NF is defined as any functional imaging technique that derives a real-time signal from an MRI scanner that keeps pace with data acquisition.^1,2^ Crucially, due to its high spatial resolution, rtfMRI-NF allows for precise targeting of specific brain regions and networks, identified using either structural or functional brain region localizers. The blood-oxygen-level-dependent (BOLD) signal from one or more brain region or network is usually presented through a visual feedback interface, with the participant then tasked with increasing or reducing the signal intensity (eg, by changing the level of a gauge or the size of a picture). The efficacy of the process can be assessed in real time by observing the regulation of signal intensity, as well as later through off-line functional analyses of signal intensity in the target region and its comparison with the signal intensity in control regions or during periods of rest.

 rtfMRI-NF has been used for a range of nonclinical and clinical applications both as a research tool and as a potential clinical intervention. In clinical populations, including those diagnosed with schizophrenia^3^ (not specific to hallucinations), major depression,^4,5^ and neuralgia,^6^ rtfMRI-NF has been shown to have treatment potential with no or minimal side effects. Recently, a few studies have used rtfMRI-NF to understand the neural mechanisms that underlie auditory-verbal hallucinations (AVH) in individuals with schizophrenia.^7–11^ This is an emerging area of research that allows for both the understanding of neural mechanisms (allowing these to be causally tested) and for reducing symptom severity (a potential clinical intervention). Such applications, however, require the identification of neural target mechanisms, ie, brain regions and/or networks, based on existing neurocognitive models of AVH.

 In this article, we summarize and review existing mechanistic models of AVH to identify feasible neural targets for the application of rtfMRI-NF as a potential tool for both research and treatment. We then consider the methodological issues and ethical implications relating to the use of rtfMRI-NF for AVH in individuals distressed by their experiences. The theoretical frameworks we discuss pertain specifically to the auditory-verbal domain and often in individuals diagnosed with schizophrenia; therefore, they are limited in the sensory modality and clinical disorders. Whilst a broader discussion is needed regarding the experience of hallucination in other modalities and diagnoses, this is outside the scope of the current review. This article emerges from an International Consortium on Hallucination Research (ICHR) working group and was, in part, presented at the 2019 biannual meeting.

## The Use of rtfMRI-NF to Test Mechanistic Models of AVH

It is important to broadly consider how cognitive models of AVH map onto functions such as language (a core feature of AVH), memory, and higher-level processes that allow us to dissociate between internally and externally generated thoughts and memories. For example, activities in speech and auditory regions/networks might relate to the loudness or physical qualities of AVH.^12^ On the other hand, engagement of cortical midline regions, thought to be important for self-referential processes^13^ and reality monitoring,^14^ might be associated with cognitive or metacognitive aspects of the experience, such as forming beliefs about whether a percept is real or imagined. A number of functional imaging studies have begun to explore the role of the brain’s default-mode network (DMN) in the experience of AVH.^15,16^ Here, we provide a brief overview of cognitive models that have provided mechanistic targets for rtfMRI-NF studies aimed at causally testing these models and examining their potential for future therapeutic interventions.

 Arguably, the most influential cognitive model of AVH is the inner speech model.^17^ This model proposes that AVH are the result of one’s own inner verbal thoughts (a kind of self-dialogue) being misattributed as external and perceived as external or alien (ie, emanating from someone else). Inner speech models of AVH have also been supported by neuroimaging findings. Whilst at a neural level, AVH are associated with activity in a network of brain regions,^18^ the most robust and replicated finding appears to be elevated and/or altered cortical activity in speech and language regions. Using symptom-capture methods, increased activity in auditory processing areas, particularly in the speech-sensitive auditory cortex,^19–21^ is widely reported in individuals with schizophrenia when they are actively experiencing AVH.^18^ Increased auditory cortex resting activity^21^ and resting cerebral perfusion are also reported in persons with AVH.^22^ Based on these findings, Orlov et al^7^ used rtfMRI-NF to reduce auditory cortex activity in individuals with treatment-resistant AVH. It was predicted that training participants to self-regulate auditory cortex activity using rtfMRI-NF would reduce the severity of AVH. Specifically, participants were trained to downregulate superior temporal cortex activity using an rtfMRI-NF protocol over 4 MRI visits during a 2-week training period. Superior temporal gyrus (STG) activity and functional connectivity in a speech sensory-motor network were compared pretraining and posttraining. Participants successfully learned to downregulate activity in their left STG over the rtfMRI-NF training, which was associated with reduced AVH symptom severity and was specifically related to the belief about the origin of their AVH. Furthermore, the role of the STG in AVH is also supported by a very recent rtfMRI-NF study, which reports that, when trained to downregulate STG activity while listening to a stranger’s voice, individuals with psychosis managed to reduce STG activation, ignore the stranger’s voice, and decrease AVH scores in clinical assessments.^8^

 The results of the Orlov et al^7^ study are also interesting in the context of inner speech models of AVH that propose the misattribution of inner speech to an external or alien source, which can occur due to a breakdown in a physiological process known as self-monitoring.^17^ The self-monitoring model assumes that, in individuals who experience AVH, inner speech is not recognized as self-generated due to altered self-monitoring, eg, a change in the signaling between speech motor and speech sensory regions in the inferior frontal lobe and posterior superior temporal gyrus, respectively.^23,24^ Interestingly, Orlov et al^7^ also reported that, posttraining, participants showed altered functional connectivity in a speech sensory-motor network between the left STG, the left inferior prefrontal gyrus (IFG), and the inferior parietal gyrus (ie, Broca’s area). This proof-of-concept study suggests that the speech-sensitive region of the left STG is a suitable target region for rtfMRI-NF in individuals with schizophrenia and distressing AVH who do not respond to conventional treatments and that successful downregulation of left STG activity can increase functional connectivity between speech motor and perception regions.

 Another theoretical concept implicated in models of hallucinations is reality monitoring.^14^ Reality monitoring refers to the cognitive processes we use to distinguish internally generated experiences from those perceived in the external world.^25^ Behaviorally, it has been shown that individuals with schizophrenia and hallucinations (usually AVH but sometimes also visual hallucinations) show reality-monitoring alterations compared with healthy volunteers and individuals without hallucinations.^24^ Whilst a number of brain regions have been linked with reality-monitoring ability,^25^ the medial prefrontal cortex (mPFC) appears to be particularly associated with differentiating between internally and externally generated information.^23–25^ This is consistent with previous findings indicating the involvement of anterior mPFC in the retrieval of self-referential information^26,27^ and in other introspective or internally generated processes. Functional neuroimaging studies have revealed that individuals with schizophrenia show differences associated with reality-monitoring changes in the anterior mPFC.^28^ Notably, the observed reduction in mPFC activity appears specifically related to reality-monitoring performance rather than an element of more general cognitive dysfunction, such as working memory.^29^ This suggests that reality monitoring might be a distinct neurocognitive indicator in schizophrenia.

In an experiment using rtfMRI neurofeedback, undertaken to gain causal evidence for the involvement of mPFC in reality monitoring, healthy volunteers received either active rtfMRI-NF from the paracingulate region of the medial prefrontal cortex or sham training based on randomized signal. After training, Active-group participants showed improved reality-monitoring accuracy for imagined items, a behavioral effect not exhibited by the Sham group nor observed for item-recognition memory. Active neurofeedback was also associated with increased midline functional connectivity between paracingulate cortex and the precuneus, a functional network connection shown to be diminished during reality monitoring in participants with schizophrenia with hallucinations (Garrison et al, in preparation; preprint accessible at https://doi.org/10.1101/2020.05.19.103572). These findings are broadly consistent with previous case-study work in individuals with schizophrenia and AVH, which found that rtfMRI-NF training targeting the anterior cingulate cortex (ACC; also a cortical midline region) can also have beneficial therapeutic effects.^9^ Taken together, these findings suggest that reality monitoring may be causally supported by activity and functional connectivity within cortical midline and sensory brain regions and that these can be altered through neurofeedback training to improve individuals’ reality-monitoring ability.

 The mPFC and the paracingulate cortex are parts of a wider functional brain network known as the DMN.^30^ Altered DMN dynamics have also been associated with specific psychotic symptoms. Northoff and Qin^31^ propose that AVH in schizophrenia may be caused by altered resting-state activity in the DMN and in the auditory cortex, possibly explaining the often self-reflective nature of auditory hallucinations. Neuroimaging studies have reported altered DMN activity and connectivity in individuals diagnosed with schizophrenia who experience AVH. A study by Jardri et al^32^ used fMRI to “capture” neural activity during hallucinations in adolescents with a brief psychotic disorder. Whilst primary sensory cortex activity was shown to be associated with the increased vividness of the hallucinatory experiences, disengagement of the DMN was concomitant with AVH. Dynamic causal modeling using fMRI data further confirmed this finding, showing the complex interaction occurring between salience, default-mode and executive networks at different stages of the experience.^18^

 Accordingly, Zweerings et al^10^ used rtfMRI-NF to further investigate the role of the DMN in AVH in individuals with schizophrenia and healthy participants. Participants underwent 2 days of rtfMRI-NF training targeting nodes in the left-hemispheric language network, including the IFG and posterior STG superior. Participants learned to downregulate and upregulate their brain activation in the designated target regions using rtfMRI-NF. Resting-state measures of activity and connectivity in the DMN were also acquired. Zweerings et al reported that coupling between nodes of the language network and DMN selectively increased after downregulation as compared to upregulation rtfMRI-NF. Network analyses revealed more pronounced increases in functional connectivity between nodes of the language network and DMN in individuals with schizophrenia compared to healthy participants. Moreover, improved well-being 4 weeks after rtfMRI-NF training predicted increased functional network coupling. Another very recent study by Bauer et al^11^ provided supporting evidence for enhanced modulations between DMN and the task-positive central executive network as one of the key functions of rtfMRI-NF in treating distressing AVH.

 Currently, rtfMRI-NF is not offered as a routinely available clinical treatment for AVH. Whilst this is mainly due to issues with logistics and cost, the scarcity of empirical evidence from studies employing this method has also prevented the use of rtfMRI-NF. The 6 recent studies mentioned in the section above nonetheless serve as a promising beginning, offering proof-of-concept, which may ultimately pave the way to therapeutic use.

## Methodological and Feasibility Issues Around the Use of rtfMRI-NF

### Standard rtfMRI-NF Procedure

**Fig. 1.**
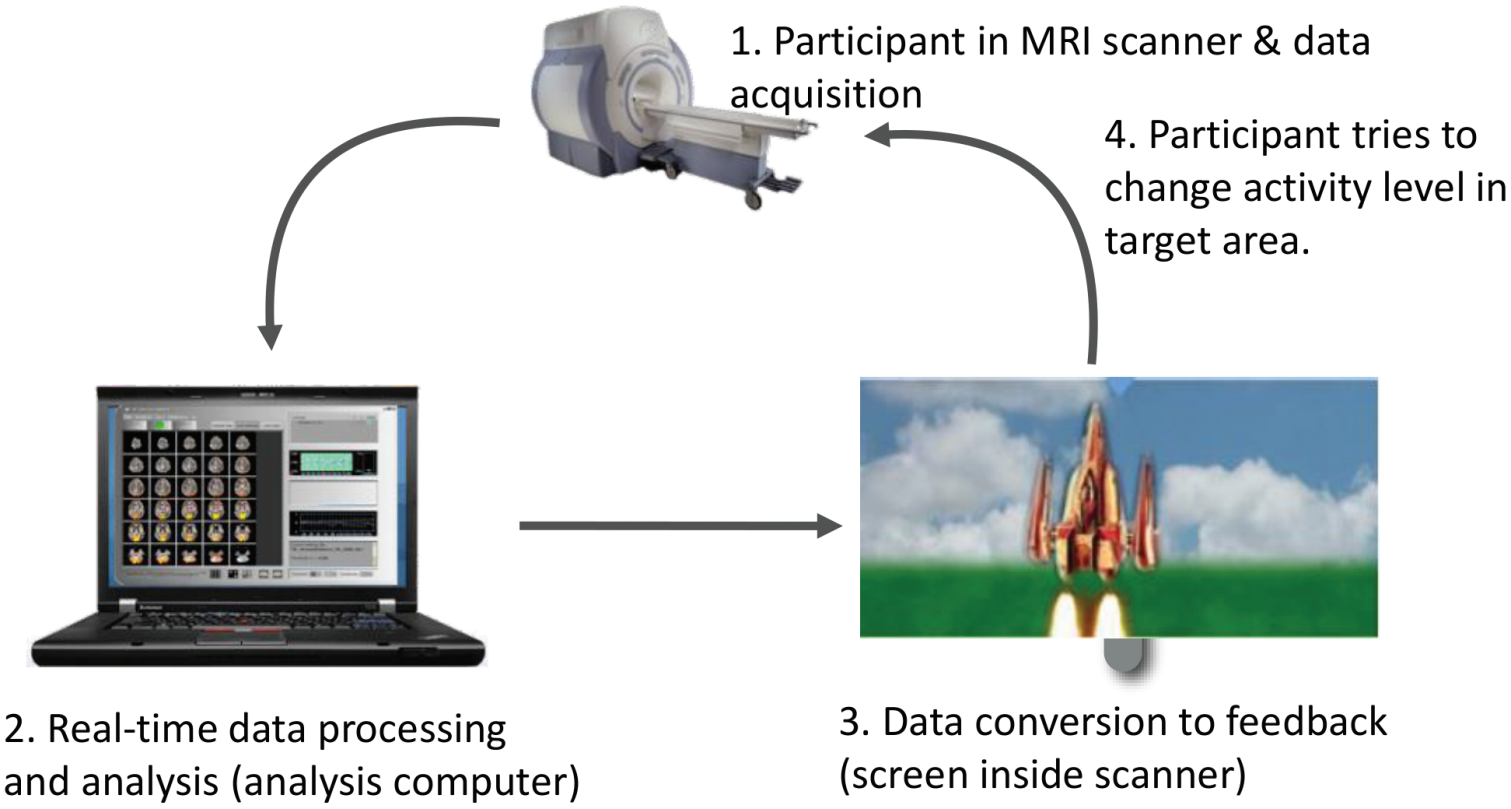
Schematic showing a typical rtfMRI-NF setup. Reproduced and modified with permission from Professor David Linden, Cardiff University, UK/Maastricht University, The Netherlands. rtfMRI-NF, real-time functional magnetic resonance imaging neurofeedback.

In a typical rtfMRI-NF protocol^33^ (figure 1), researchers start by explaining the procedure to participants, administer consent forms, explain the hemodynamic time lag (usually around 6–10 s), and may suggest a strategy to help the participants modulate the BOLD signal of interest.^34^ Participants lie in an MRI scanner and look at a display screen. It is worth noting that feedback does not have to be presented via the visual domain and can in fact use any sensory modality, although visual feedback is the most commonly adopted method for AVH. After a structural brain scan, researchers use a localizer procedure to identify voxels from which they will provide feedback (the target region of interest; ROI). Participants then usually undergo a number of neurofeedback runs wherein they view a simplified representation of brain activity originating from the ROI, such as using a thermometer-style bar graph, although more sophisticated visual feedback interfaces are beginning to be more widely used. These runs generally last between 5 and 10 min and alternate between approximately 20 and 60 s of active blocks, when participants attempt to modulate the visual feedback, and rest blocks, when participants refrain from attempting to modify the BOLD signal. Participants must hold still and maintain their head position throughout. A “transfer run” also takes place during the last scanning visit. This is identical to training runs but without feedback, which allows the assessment of the overall success of the training (retention of learning). Depending on the exact experimental design, control groups generally receive a sham neurofeedback signal or a signal from a control brain region. An average scanning session lasts 1–2 h but training may occur over multiple days.

### Choice of Localizer: Structural, Functional, Multivariate Pattern Classifiers

**Fig. 2.**
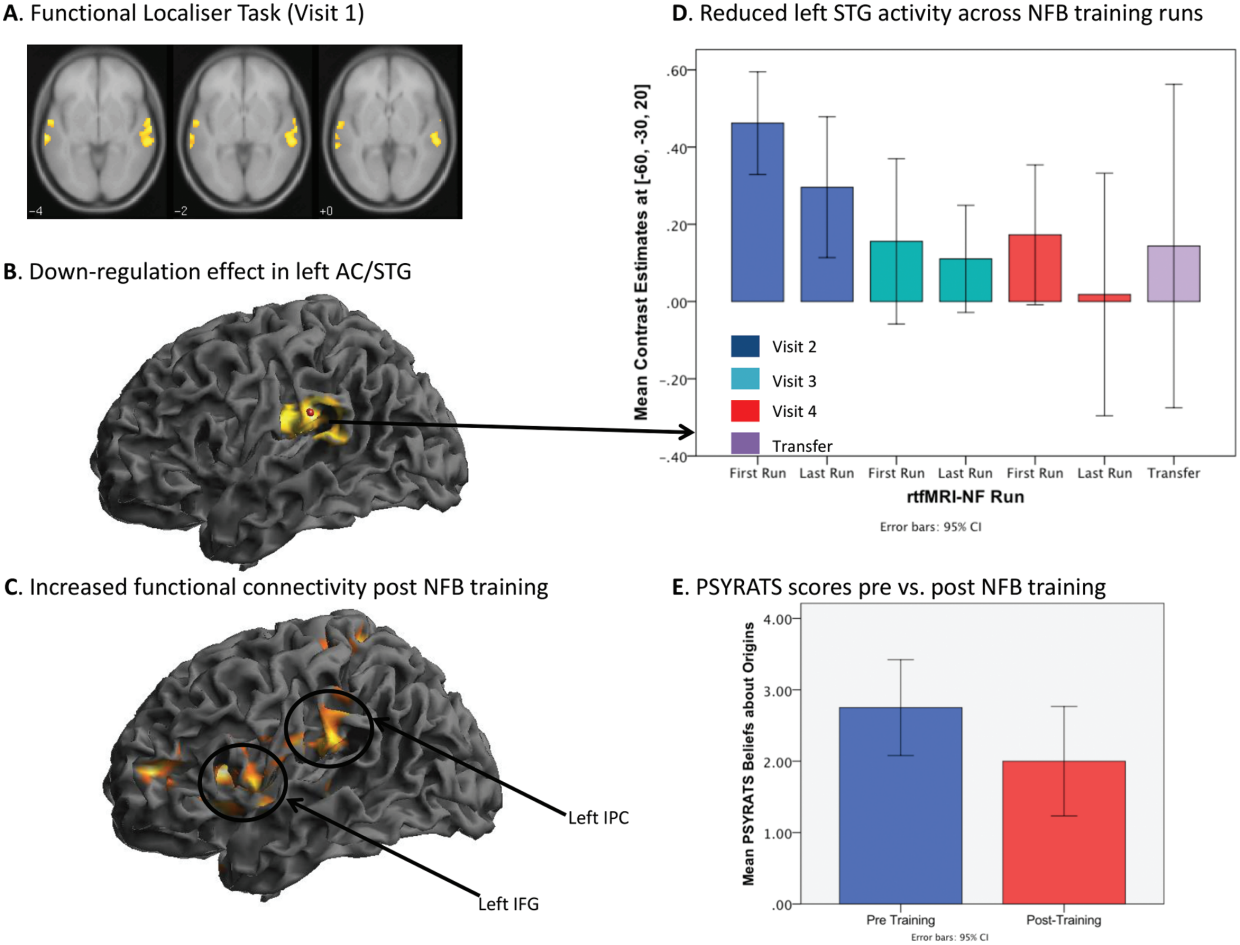
Schematic showing the effects of rtfMRI-NF training on key brain regions and on symptomatology. AC, anterior cingulate; STG, superior temporal gyrus; IPC, inferior parietal cortex; IFG, inferior frontal gyrus; PSYRATS, Psychotic Symptoms Rating Scale; rtfMRI-NF, real-time functional magnetic resonance imaging neurofeedback.

Usually, either a structural or functional localizer is used for the rtfMRI-NF signal. For a structural localizer, the rtfMRI-NF signal is derived from a brain region localized using a high-resolution structural MRI scan data and a brain atlas, most likely focusing on brain regions that show altered activity during AVH. Suggestions would include cortical areas involved in auditory processing (eg, STG; see figure 2) for AVHs or areas that may regulate hallucinatory activity, such as the ACC and/or paracingulate regions. This is the easiest localizer method to implement with the most experimental data to support its use. Its efficacy will depend on strong theoretical support for the choice of location and its functional anatomy and minimization of individual differences in location identification.

 For a functional localizer, the subject usually undertakes a functional task in the scanner (prior to rtfMRI-NF training) to activate areas or networks thought to underlie a cognitive, behavioral, or psychological process. The identified ROI(s) is then used for subsequent rtfMRI-NF training. Possible sites of interest are similar to those that could be identified for the structural localizer above and should be well validated from earlier studies. This method of identifying the ROI is technically more complex than for the structural localizer, where univariate fMRI analysis of the difference between the average BOLD signal in the target region during the task needs to be compared with a baseline signal. Issues here include the need to manually specify the statistical thresholds to provide a cluster of the required size (which is likely to vary across participants) to act as the ROI. Also, there is the choice of whether the ROI is redefined for each rtfMRI-NF scanning session, or an initial mask is defined during the first session to save scanning time subsequently, with a careful realignment of images from the different sessions. The goal for both the structural and functional localizers is for participants to learn to regulate neural activity within these regions.

 For a multivariate localizer, instead of basing the ROI on trait markers of hallucinations identified using structural or functional localizers, the ROI can be defined using state markers that correlate with the experiential states of interest (eg, AVH). In this case, the objective is to train the participant to self-regulate the pattern of activity across brain areas that reactivate during certain altered states. A multivariate classifier is then used to define the ROI for the rtfMRI-NF. A training scanning session is used to allow the optimal classifier to be built on the training data set for which the symptomatic and asymptomatic periods of interest have been identified. A second validation session is then needed to test the performance of the classifier, before commencing the rtfMRI-NF therapeutic sessions.

### Choice of Control/Sham Condition for a Blinded Study

Several different approaches have been adopted for the choice of a control condition, which provides a baseline for the determination of the efficacy of the rtfMRI-NF intervention:

1. Taking a sham feedback signal from a previous participant—this can lead to frustration as participants realize the noncontingency of the feedback with the possibility of the participant becoming unblinded. People might react very differently in this respect and the reaction is methodologically difficult to study and monitor.
2. Using a randomized sham signal of similar intensity to active condition—disadvantages as for (1).
3. Using the inverse signal of interest—disadvantages as for (1) and unethical when used for hallucination intervention or in cases where a signal change in a certain direction could, theoretically, exacerbate a distressing experience.
4. Participants undertake a mental task outside the scanner related to the activity that is occurring in the scanner, eg, imagining hearing a voice or seeing a vision. This saves on scanner costs but is an unsatisfactory control as participants are not exposed to scanner conditions.
5. Taking neurofeedback signal from a control brain region unrelated to hallucinations—this is the preferred option, but the choice of control region is critical, eg, visual cortex may be a suitable control region for an intervention focused on AVH but would not be suitable for visual hallucinations.

## Methodological and Logistical Issues Around the Use of rtfMRI-NF for Individuals With AVH

In addition to the standard methodological issues and approaches related to rtfMRI-NF studies, there are a number of issues pertaining to its use as a therapeutic intervention for individuals with AVH. We have summarized these and offered recommendations in Textbox 1 below.

**Textbox 1. Methodological Recommendations According to Study Design**

- *Proof-of-concept and optimization studies:* First, preliminary research shows that rtfMRI-NF has potential value for reducing the intensity, stress, or disruption caused by hallucinations. However, the sample sizes so far in these preliminary and pilot studies have been small. Whilst these studies have been helpful in addressing proof-of-concept issues, far more experimental work is needed to establish the optimum and most efficacious experimental designs, explore individual differences in efficacy, and determine whether rtfMRI-NF results in durable clinical effects. Little has been done to address these questions so far, despite its being an essential requirement of a viable therapeutic intervention.
- *Cost vs therapeutic-economic benefit*: MRI scanner time costs can be very high and would be even greater for protocols requiring repeated scanner visits, which are often needed for rtfMRI-NF training protocols. Such costs should, in turn, be balanced against potential cost savings in relation to other clinical approaches. The cost benefits of rtfMRI-NF as an adjunct therapy also need to be evaluated.
- *Randomized Controlled Trials (RCT)*: As with all new therapeutic interventions, successful RCTs are needed before a therapeutic intervention for distressing hallucinations could be widely implemented. Ideally, in effective RCTs, control groups receive a highly comparable treatment that omits the active ingredient or mechanism of action purported to drive improvement, and neither participants nor experimenters can identify who receives the active vs placebo treatment (double blinding). Undertaking such a double-blinded RCT using rtfMRI-NF is challenging, however, due to the difficulties in the blinding of researchers who are often necessarily aware whether a participant is receiving an active or sham rtfMRI-NF signal. However, 7 successful double-blinded rtfMRI-NF studies have now been undertaken to date,^4–6,35–38^ albeit not for the study of distressing hallucinations. One particular strategy that allows for double blinding is to have the member of the study team who interacts with the participant kept blind from the experimental condition. The researcher(s) running the feedback protocol are blinded as to the participant group.
- *Scanner-acceptability and imaging requirements*: Some individuals will be unsuitable for the procedure due to metal in their bodies, claustrophobia and anxiety, residential distance from a scanner location, lack of motivation to undertake the procedure due to anhedonia, depression, or cognitive impairment (reduced ability to self-reflect). Furthermore, there may be additional issues for use of a rtfMRI-NF technique with clinical populations. Movement and respiration can both cause changes in the BOLD signal (artifacts) and the potential for these artifacts are during rtfMRI-NF training protocols relative to standard MRI procedures.^34^ As such, instructions to limit movement are crucial, which may be a particular issue if participants are actively experiencing hallucinations and/or anxiety. The very limited experimental evidence using multivariate classifiers within participants during hallucinatory states suggests that they can be used to detect the occurrence of AVHs with greater than 71% accuracy, regardless of real-time artifacts.^39^ As yet, however, no studies have used multivariate classifiers in an rtfMRI-NF protocol to construct the feedback signal for training. For this approach to be successful in the treatment of distressing hallucinations, two additional constraints are required in addition to those using structural and functional localizers:

1. Hallucinations must occur several times during the rtfMRI-NF scanning session so that the classifier can be trained, perhaps limiting the suitability of some individuals.
2. It must be possible for the classifier to dissociate symptomatic from asymptomatic periods, eg, on the basis of data analysis and clinical interviews.

## rtfMRI, Distress, and the Need for Treatment

As is likely the case for any emerging technology, the use of rtfMRI-NF has implications for how clinicians and the general public view mental illness, its nature, and its (need for) treatment. As mentioned in previous sections, the objective of rtfMRI-NF is to train people to modulate their own neural activity via feedback from their BOLD responses. As part of a therapeutic application, individuals are trained to change or modulate BOLD response believed to be related to an aberrant or pathological neurocognitive process that underlies the experience of hallucinations themselves (eg, perceptual expectations or speech monitoring). However, it is now well-established and widely accepted that AVH occur, sometimes with the same frequency and intensity, in nonclinical individuals and populations with no need for psychiatric care or intervention.^40^ Further, whilst outside the scope of this article, AVH show considerable phenomenological diversity,^41^ which may call for tailored approaches.

 Distress associated with hallucinations is an important trigger for seeking treatment, and rtfMRI-NF may need further development to address that aspect. If rtfMRI-NF receives further empirical support, clinicians should inform individuals suffering from hallucinations about this possibility, in addition to other existing treatments. Thus, people with AVH should always be fully informed and supported to make their own decisions regarding treatment. Indeed, rtfMRI-NF as a therapy for hallucinations is strongly dependent on the active participation of the individual. This may have the additional benefit of empowering people with AVH by putting them center stage in gaining control over processes in their brains.

## Future Directions for Research and Practice

There is great potential for rtfMRI-NF in hallucination research, both in terms of fundamental research that allows investigators to test neurocognitive and pathophysiological models and the development of novel interventions and therapies that could help people who experience distressing hallucinations. A pertinent question is the durability of clinical efficacy, which needs to be assessed via longer-term follow-up studies (to date, no study has addressed this specifically for AVH). Issues with cost and portability may be addressed in the near future by the transfer of fMRI procedures to lighter devices using almost-similar hemodynamic signals like functional near-infrared spectroscopy. Whilst there is great potential, there are also a number of unique challenges. For example, individuals with schizophrenia (a likely beneficiary group) also experience marked deficits in cognitive and executive function that can affect learning, a crucial process during rtfMRI-NF training.

 Moreover, impairments are not necessarily limited to executive functions (attention, concentration, working memory, engagement with task, etc.) alone but are likely to involve prereflective subtle cognitive changes present throughout the course of a psychotic illness, for instance, those captured by the basic self-disorder framework.^42,43^ Basic self-disorders directly impact individuals’ fundamental self-awareness and frequently damage their ability to reflect upon or even feel in control of their own mental functions.^44^ Given that the brain regions involved in these disorders are extremely widespread and diverse, it might be more useful for future research to target overall spatiotemporal dynamics rather than specific brain regions or cognitive function.^45^

 One way to achieve this might be combined (bimodal) real-time EEG-fMRI neurofeedback where the participant wears an EEG cap inside an MRI scanner and EEG recordings are performed concurrently with fMRI data acquisition.^46^ EEG neurofeedback has its own advantages in the temporal dimension (eg, the different frequencies used in EEG) and may provide crucial insights in the neural dynamics of AVH and related experiences that are easily missed by the sluggish hemodynamic response associated with fMRI. This combined method has been performed in healthy volunteers^47^ and small groups of participants with neurological syndromes (eg, stroke^48^) but not yet in clinical populations with schizophrenia or AVH. Therefore, a pertinent research question for the future development of neurofeedback technologies may be how to maximize the benefits of both EEG and fMRI procedures that can be used together to capture a rich spatiotemporal picture of the hallucinating brain. Similarly, an important clinical consideration is how their specific temporal and spatial properties can map onto the phenomenology of AVH (eg, via symptom-capture studies).

## Conclusion

rtfMRI-NF is a promising and innovative research tool with potential as a novel treatment for distressing hallucination. However, there are a number of developments that are still required before any such therapeutic potential can be realized. To this end, there are now internationally agreed checklists that can be used to standardize the procedures and protocols used in clinical neurofeedback.^33,49^ Although these are not specific to individuals experiencing distressing hallucinations, they provide an important basis for the development of future RCTs. Here, we have outlined a variety of potential target mechanisms for the application of rtfMRI-NF for the research and treatment of hallucination and discussed the most pertinent issues relating to the practicality of rtfMRI-NF in a clinical setting. We tentatively suggest that, whilst the logistical consideration associated with the setup of rtfMRI-NF can appear daunting at present, the approach should at least be considered as a novel and effective procedure for causally testing mechanistic hypotheses in relation to neurocognitive underpinning of hallucination.

 In terms of therapeutic applications of rtfMFI-NF for hallucinations, certain factors may best determine which individuals are most likely to respond to a rtfMRI-NF intervention. Psychotic disorders are often characterized by changes in metacognitive or self-reflective capacities. This may diminish the efficacy of interventions like rtfMRI-NF that require participants to engage, to some extent, in a self-reflective or regulatory process over their own brain activities (see self-disorders above). Therefore, clinicians and researchers must consider ethical, as well as clinical and methodological, implications. Textbox 2 presents a list of myths and misconceptions that are commonly associated with rtfMRI-NF that researchers might wish to bear in mind when communicating with participants.

**Textbox 2: Common Myths and Misconceptions About Neurofeedback**

- It involves a strong electric current and “may be dangerous.”
- It is the same as electroconvulsive therapy and “zaps one’s brain”, leading to memory problems.
- The magnetic field is “too mild” and, hence, the procedure will not affect deeper brain layers.
- Its effectiveness relies entirely on one’s willpower.
- It changes brain structure and “kills off neurons,” causing brain damage.
- It is used by “the Government” to read, steal, or modify one’s thoughts.
- It cannot be used concurrently with medication.
- It diminishes the effects of psychological (“talking”) therapies.

In sum, despite the potential difficulties in its implementation, rtfMRI-NF may still prove beneficial to individuals experiencing persistent and distressing AVH; it is also far less invasive than some of the alternatives, such as electroconvulsive therapy and repetitive transcranial magnetic stimulation, and is likely to have fewer side effects than medication. Even if the therapeutic potential of rtfMRI-NF is limited at this stage, the technique provides researchers with a tool to causally test mechanistic models of AVH, which is surely a crucial step on the pathway to the development of new interventions. Finally, there are clear challenges in the use of rtfMRI-NF for hallucinations in other modalities as their mechanistic models are arguably not as well developed as those that attempt to explain AVH. Future research should consider alternative mechanistic models that are not rooted in schizophrenia-centric research, which focuses heavily on temporal cortex activity and associated connectivity.
